# No legacy effects of severe drought on carbon and water fluxes in a Mediterranean oak forest

**DOI:** 10.1111/plb.70082

**Published:** 2025-08-10

**Authors:** S. Heinrich, X. Yu, J.‐M. Limousin, C. Werner, A. Bastos, A. Hoek van Dijke, S. Walther, J. Kroll, R. Orth

**Affiliations:** ^1^ Research Group Modeling of Biogeochemical Systems, Faculty of Environment and Natural Resources University of Freiburg Freiburg Germany; ^2^ Department of Biogeochemical Integration Max Planck Institute for Biogeochemistry Jena Germany; ^3^ Department of Ecology Universität Innsbruck Innsbruck Austria; ^4^ CEFE Univ Montpellier, CNRS, EPHE, IRD Montpellier France; ^5^ Research Group Ecosystem Physiology, Faculty of Environment and Natural Resources University of Freiburg Freiburg Germany; ^6^ Institute for Earth System Science and Remote Sensing Leipzig University Leipzig Germany; ^7^ Louis Bolk Instituut Bunnik The Netherlands

**Keywords:** drought, eddy covariance, Mediterranean forest, random forest, recovery, sap flow

## Abstract

- Severe droughts affect vegetation through several processes, such as hydraulic failure, early leaf senescence, depletion of carbon reserves, and reduced growth. These, in turn, can delay drought recovery and influence ecosystem functioning beyond the drought duration.
- The goal of this study is to investigate the direct response and physiological recovery of a Mediterranean oak (*Quercus ilex* L.) forest in southern France following the 2017 drought. We analysed eddy covariance‐based observations of gross primary productivity (GPP), evapotranspiration (ET) and tree sap flow measurements. To study drought recovery, we used a random forest regression model to predict vegetation functioning in the post‐drought years based on hydro‐meteorological conditions. Potential legacy effects can be indicated by the difference between predicted and actual values.
- The 2017 drought peaked in autumn, with the lowest soil moisture of the study period 2000–2021. Concurrently, we detected the lowest GPP, ET, and sap flow for this time of the year on record. Despite severe reductions in vegetation functioning during drought, we found no legacy effects on GPP, ET, and sap flow. This suggests that the physiological functioning of *Q. ilex* woodlands recovers rapidly and completely. We hypothesize that this fast recovery is supported by favourable pre‐ and post‐drought hydro‐meteorological conditions, as spring 2017 was unusually sunny but not water‐limited, and 2018 was the wettest year in the studied record.
- High drought resilience of *Q. ilex* forests is important in the context of anticipated increase in drought frequency and intensity under climate change. However, it remains yet to be determined to what extent the drought resilience can be sustained during potentially recurrent droughts in the future.

## Graphical abstract

A Mediterranean *Quercus ilex* forest was severely affected by a drought in 2017 but fully recovered in the following year, supported by favourable weather and growing conditions both before and after the event.

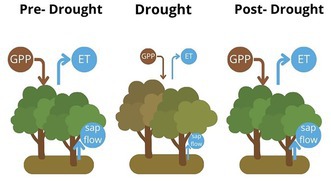

## Introduction

The intensity and frequency of droughts are expected to increase in many regions with ongoing climate change (IPCC 2023). Already in the 20th century, impacts of droughts have increased globally (Schwalm *et al*. 2017). Droughts have a strong impact on the interannual variability of global primary productivity, even compared to other extreme events (Zscheischler *et al*. 2014; Orth *et al*. 2022). Vegetation drought resilience is highly variable among different species and biomes (Vicente‐Serrano *et al*. 2013; Gazol *et al*. 2018), but studies from across biomes report droughts as an important driver of tree mortality (McDowell 2011; Schuldt *et al*. 2020; Sánchez‐Pinillos *et al*. 2022). In recent years, severe drought–heat events have caused reductions in net ecosystem productivity in most affected regions across Europe (Ciais *et al*. 2005; Bastos *et al*. 2020) or have even shifted ecosystems towards their tipping points (Haberstroh *et al*. 2022a, and this issue).

Post‐drought ecosystem dynamics have recently received increased attention (Kannenberg *et al*. 2020; Müller & Bahn 2022; Vilonen *et al*. 2022). On the one hand, several mechanisms may delay recovery, such as hydraulic failure (Choat *et al*. 2012) and depletion of carbon reserve (Mitchell *et al*. 2013). On the other hand, increased nutrient availability (Mackie *et al*. 2019) or prioritized belowground allocation (Hagedorn *et al*. 2016) can lead to enhanced ecosystem functioning after droughts (overview in Müller & Bahn 2022). Further, characteristics of the drought itself, such as drought intensity, timing, and duration (Forner *et al*. 2018; Kannenberg *et al*. 2020), and the pre‐ and post‐drought hydro‐meteorological conditions (Kannenberg *et al*. 2020) could modulate the recovery. Ruehr *et al*. (2019) established a framework that suggests broad categories of post‐stress recovery in woody plants, with fast and complete recovery being observed after mild stress intensities, while severe stress leads to impaired recovery. As a result of delayed recovery, so called “legacy effects” were found to be widespread in temperate forest ecosystems (Anderegg *et al*. 2015) and may last up to several years after the drought has ended (Yu *et al*. submitted; Müller & Bahn 2022; Vilonen *et al*. 2022).

Drought recovery studies often consider single ecosystem response variables, such as tree growth (Anderegg *et al*. 2015), satellite‐based greenness (Wu *et al*. 2022), and gross primary productivity (GPP) (Pohl *et al*. 2023). Few studies have included and compared multiple variables, such as GPP, stem growth, and leaf‐level photosynthesis (Kannenberg *et al*. 2019; Werner *et al*. 2021). Ruehr *et al*. (2019) highlight that different ecophysiological traits differ in their recovery duration, with leaf water potential typically recovering quickly, while hydraulic conductance and leaf gas exchange may take longer to recover. Therefore, there is still a high uncertainty on how drought recovery dynamics and legacy effects compare across variables. Additionally, in the case of sap flow, observational evidence on the recovery rate and the extent of potential legacy effects is still sparse (Forner *et al*. 2014; Hesse *et al*. 2023). Sap flow is less commonly considered in long‐term ecosystem monitoring as temporal sampling errors can affect measurements (Moore *et al*. 2010). Here, we propose a novel approach for processing long‐term sap flow measurements that accounts for both sensor replacements and signal loss. Sap flow observations could complement our understanding of drought recovery as they indicate the functioning of the hydraulic system of trees (Choat *et al*. 2012).

In order to investigate the drought response and recovery across multiple variables, we consider sap flow of several trees alongside GPP and Evapotranspiration (ET) from eddy covariance measurements. Thus, in this study we focus on physiological parameters and the response and recovery of carbon and water fluxes but do not further evaluate the recovery in terms of morphological changes, such as leaf size or stem growth. We evaluate drought recovery using an approach described by Yu *et al*. (2022), where a random forest model is trained to learn the relationship between hydro‐meteorological conditions and ecosystem response during non‐post‐drought years. Applying this model for predicting ecosystem variables after the drought allows us to estimate potential legacy effects by comparing predicted with actual values. We relate the results to the context of preceding and subsequent meteorological conditions, as well as to observed vegetation greenness.

The Mediterranean basin was affected by a severe drought in 2017 that was associated with tree mortality in Spain (Hartmann *et al*. 2022). In this study, we focus on a Mediterranean forest in southern France that is dominated by *Quercus ilex* and has been subject to several studies on drought effects. These studies highlight, for example, the importance of drought timing on stem growth (Lempereur *et al*. 2015) and on the annual net ecosystem productivity (Allard *et al*. 2008). The main research question of this current study is: How did the 2017 drought, the most severe drought in the recorded data, affect ecosystem functioning in a Mediterranean *Q. ilex* forest, and did sap flow, GPP and ET fully recover in the post‐drought years?

## Data and Methods

### Study site and species description

The study site is located in southern France near Puéchabon (43°44′30″ N, 3°35′40″ E). The climate is Mediterranean, with a mean annual temperature of 13.48°C and a mean annual precipitation of 930 mm between 1988 and 2017. The soil is very heterogeneous, rocky and of hard Jurassic limestone origin. An overview of stand characteristics is provided in Table 1, and a detailed site description can be found in Limousin *et al*. (2009).

**Table 1:** Overview of stand characteristics. Information is based on Limousin *et al.* (2009) and Ruffault *et al.* (2023).

Stand characteristics	
dominant species	holm oak (*Quercus ilex*)
mean annual temperature	13.48°C (between 1988 and 2017)
mean annual precipitation	930 mm (between 1988 and 2017)
stand age	trees re‐growing since 1942 (previously managed as coppice)
top canopy height	5.5 m (in 2016)
tree diameter	75% of trees between 4 and 10 cm
stand density	4544 (± 719) stems ha^−1^ (in 2016)
soil characteristics	Shallow bedrock, heterogeneous and rocky soil originating from hard Jurassic limestone
volumetric fractional content of stones	0.75 for the top 0–50 cm and 0.90 below

The dominant tree species is *Quercus ilex* L., an evergreen oak, that is considered as a “keystone species” in parts of the Mediterranean basin (Barbeta & Peñuelas 2016). *Q. ilex* has various morphological and physiological adaptations to water‐limiting conditions (Barbeta & Peñuelas 2016) and might replace less adapted species with an increase in drought intensities (Aguadé *et al*. 2015). In the current literature, there are contrasting statements about the drought tolerance and water use strategies of oaks (Novick *et al*. 2022). In particular, for *Q. ilex* some literature concludes that it might have a strongly water conservative strategy, being one of the “most isohydric species of the genus” (Martínez‐Vilalta *et al*. 2014; Barbeta & Peñuelas 2016). On the other hand, studies have found a more anisohydric behaviour of *Q. ilex* compared to other genera (Aguadé *et al*. 2015). Long‐term water potential observations supported anisohydric behaviour, with a turgor loss point of −3.67 MPa that is regularly exceeded (Limousin *et al*. 2022). It has been shown that the strict alignment to either isohydric or anisohydric behaviour does often not hold and, for example, for *Q. suber*, a closely related Mediterranean oak, a shift from more isohydric to anisohydric behaviour has been observed in response to environmental changes (Haberstroh *et al*. 2022b).

Growth and productivity of *Q. ilex* show a bimodal pattern, with a peak in the first half of the year and a second peak in autumn (Barbeta & Peñuelas 2016). Thus, it shows little response under moderate drought stress during hot and dry summer periods (Barbeta & Peñuelas 2016). However, under severe drought stress or if a drought occurs in the wet season, negative drought effects have been observed (Forner *et al*. 2018; El‐Madany *et al*. 2020; Limousin *et al*. 2022). For example, increased leaf shedding reduces non‐structural carbon reservoirs (Barbeta & Peñuelas 2016) and the photosynthetic leaf area with potential effects on ecosystem functioning beyond the drought period (Limousin *et al*. 2009; Ruffault *et al*. 2023). At this site, all *Q. ilex* trees have been regrowing since 1942 after the stand had been managed as a coppice for several years (Limousin *et al*. 2009).

It should be noted that droughts occur repeatedly in the typical Mediterranean climate at Puéchabon within our study period, for example in 2006 and 2016. However, in this study we focus only on the 2017 drought, as this year showed the strongest meteorological drought conditions along with structural damage caused by severe tree water stress (see Section 3.1). Nevertheless, preceding dry conditions in 2016 could have affected the resilience of the ecosystem during the drought in 2017.

### Data

This study focuses on the period 2003–2021 and uses daily averages of measurements of several ecosystem variables.

Sap flow was measured at half‐hourly intervals with Granier's Thermal Dissipation Probes (TDP) (Granier 1985) using 20‐mm long needles with a vertical distance of 10 cm. All sensors were installed on the north side and shielded using aluminium protection. The half‐hourly mean records were stored on data loggers. For a more detailed description see Limousin *et al*. (2009). Each tree has been equipped with two or three sap flow sensors within the study period. These sap flow sensors are known to show decreasing signal strengths when measuring over long time periods (Moore *et al*. 2010). In order to address the signal loss and sensor replacements, we establish a new processing approach (see Section 2.3.1). Long‐term measurements of six trees are available. The data for one tree were excluded from the analysis due to implausible dynamics that differed from the responses of other trees, pointing towards sensor problems, as they do not align with the observed meteorological conditions.

Eddy covariance (EC) measurements of ET and GPP were obtained at the top of a 12 m‐high tower, which is about twice the height of the tree canopy at the site. From 2003 to 2013 the EC set‐up consisted of a three‐dimensional sonic anemometer (Solent R3; Gill Instruments, Lymington, UK) and a closed path infrared gas analyser (IRGA, model LI 6262; Li‐Cor Inc.) as described in Allard *et al*. (2008); from 2014 to 2021 the EC set‐up followed the ICOS protocols (Rebmann *et al*. 2018) and consisted of a HS‐50 sonic anemometer (Gill Instruments) with a Li‐Cor 7200 IRGA (Li‐Cor). Data acquisition was obtained at a rate of 20 Hz and processed with the EddyPro software (Li‐Cor) following FLUXNET and ICOS recommendations (Rebmann *et al*. 2018) for data quality check and filtering. Data gap filling at the half‐hourly scale was performed and GPP was derived using the night‐time partitioning method of Reichstein *et al*. (2005) in the REddyProc R package (Wutzler *et al*. 2018).

Hydro‐meteorological conditions were measured on half‐hourly scale at the EC tower. The Soil Water Content was calculated using the process‐based vegetation model SIERRA (Mouillot *et al*. 2001) that computes the difference between precipitation as the only water input and the sum of output fluxes (evapotranspiration, interception, surface runoff, and deep drainage) on a daily time scale (Cabon *et al*. 2018). The model included site‐specific parameters, including a rooting depth of 4.5 m (Rambal *et al*. 2003), a stone fraction and fine root distribution of 70% in the upper 1 m, and the other fine roots up to 4.5 m depth (Cabon *et al*. 2018).

In addition to the measurements collected on site, satellite‐derived enhanced vegetation index (EVI) data from Moderate Resolution Imaging Spectroradiometer (MODIS) was employed, as retrieved from FluxnetEO dataset (doi: 10.18160/XTV7‐WXVZ). The data represent an area of 1 km around the site on a daily scale and is quality‐checked and gap‐filled by Walther *et al*. (2022). A significant positive trend in EVI was detected and removed using a linear model to avoid potential drought effects being partly masked by long‐term greening.

### Data pre‐processing

For most of the variables used in this study, sensors have been replaced during the study period. Furthermore, in the case of sap flow, measurements are available from several individual sensors for the same tree. Therefore, we pre‐process the data as described below to: (i) ensure the long‐term consistency of the observation time series, and (ii) make the sap flow time series more comparable with the EC measured GPP and ET by combining multiple sap flow sensors from individual trees. Note that as a consequence of the data processing the resulting absolute values are no longer physically interpretable, while the temporal dynamics become consistent across the study period.

#### Sap flow

So far, there is no common approach established for both combining different sensors and accounting for a decreasing signal strength of long‐term sap flow measurements (Poyatos *et al*. 2021). Some studies combine different sensors based on a linear relationship between sensors (Rascher *et al*. 2011). However, this approach is not possible in our case due to progressing sensor degradation.

**Fig. 1:**
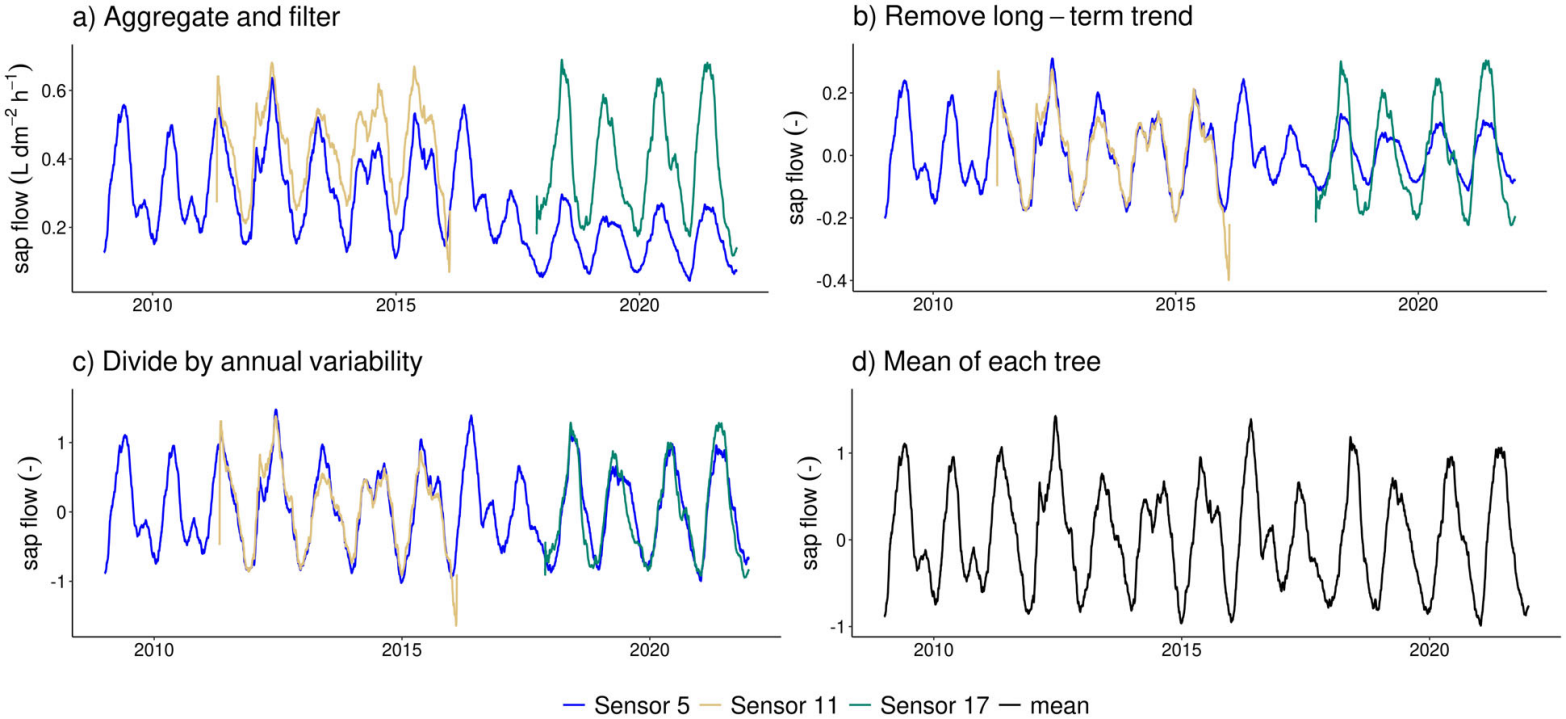
Sap flow data processing. Steps a), b) and c) are applied to time series of each sensor individually. Lines in different colours represent different sensors at the same tree. Only part of a time series of an example tree is shown here. A 3‐month moving average is applied in this figure for better readability.

To address these aspects, we developed a novel stepwise data processing approach for the sap flow data from individual sensors of each tree (see Fig. 1). It should be noted that while the sap flow data pre‐processing accounts for the effects of sensor degradation, we do not perform a gap‐filling.

- Aggregate and filter: Data are being aggregated from half‐hourly to daily scale using the daytime mean (based on daytime flag in EC data). Daily values are computed if at least 70% of the respective half‐hourly values are available. Years are considered in our analysis if at least 40% of the daily values are available. Daily values are considered as outliers and removed if they are more than three standard deviations away from the annual mean. This outlier filtering removed less than 1% of daily sap flow values, a maximum of 13 days were removed within individual sensors.
- Remove long‐term trend: Any long‐term variability is extracted using a loess fitting (stl function from package stats in R, using 3‐year span of the loess window for trend extraction) and subtracted the resulting trend from the original time series.
- Divide by annual variability: To adjust for changes in variability over time caused by decreasing sensor signal strength, an annual standard deviation of sap flow is determined with a 3‐year moving window that takes into account neighbouring years to capture long‐term variability changes. Then sap flow is divided by annual values calculated with the moving window.
- Mean of each tree: Daily means are calculated across all available sap flow sensors.

#### Eddy covariance data

In order to compensate for the effect of different EC set‐ups, we separately normalize the time series before and after 2013 by subtracting the long‐term mean and dividing by the standard deviation. The resulting normalized time series are then connected. This processing procedure is applied to both GPP and ET from EC data.

### Analysis of drought impacts and recovery

To quantify direct impacts of the drought, we compare sap flow, GPP and ET in 2017 with long‐term averages from 2003 to 2021. Mean values for the whole year 2017 as well as first and second half of the year are computed and compared with respective mean values of all other years. To be comparable across variables, we compare the differences between mean values of 2017 and long‐term mean with the standard deviation of each variable.

We study drought recovery and potential legacy effects during 2018 and 2019 as (i) drought legacy effects have been observed several years after droughts (Müller & Bahn 2022), and (ii) 2018 was an extraordinary wet year such that potential legacy effects might be masked and are then only observed later in 2019.

To characterize the drought recovery dynamics, we follow a residual approach of Yu *et al*. (2022). Post‐drought measurements are compared with predictions from a statistical model which is trained without post‐drought information. In particular, a random forest regression model (package randomForest in R) learns the relation of hydro‐meteorological variables and the target variable (i.e. sap flow, GPP or ET) in the training data. We include soil water content (SWC), vapour pressure deficit (VPD), shortwave radiation (SW_IN), air temperature (TA), wind speed (WS), and precipitation (precip) as predictors. The training data include all years, except for the 2 years following the drought, which are considered as the post‐drought period. Thus, the model cannot learn mechanisms of potential drought legacy effects.

The model then predicts the target variable in the post‐drought period, using the hydro‐meteorological conditions within that period as predictors. Then, model residuals are calculated by the difference between measurements and predicted values. These model residuals can be interpreted as legacy effects, which can be positive or negative as indicated by higher or lower observed values compared to predicted values, respectively. In the case of a full recovery, model residuals would be close to zero.

Additionally, to calculate model uncertainties, a leave‐one‐out approach is performed (Yu *et al*. 2022). The model is trained several times and each time the post‐drought years and one additional non‐post‐drought year are excluded from the training. Then the target variable is predicted for the considered non‐post‐drought year based on respective hydro‐meteorological conditions. The differences between predicted and observed values indicate the uncertainty of the random forest approach. We quantify this through the 25th–75th and 5th–95th percentile ranges across residuals of all non‐post‐drought years. Potential legacy effects in the post‐drought years are compared with this uncertainty range to assess their significance, and to distinguish recovery dynamics from natural variability and model uncertainty.

This approach is applied to sap flow using data of individual trees separately. Additionally, all trees are included in the same model using their tree ID as an additional predictor to obtain ecosystem‐scale legacy effects. Model performance as obtained from Out‐of‐bag scores achieves comparable results for individual trees and aggregating trees, thus the results for both approaches are considered.

## Results and Discussion

### Drought conditions and ecosystem response

The drought event in 2016 and 2017 was described as the ‘most severe event in western Europe since at least 1979’ [until 2017] (García‐Herrera *et al*. 2019) and showed unusual spatial patterns affecting both northern and southern Europe (Ayarzagüena *et al*. 2018; García‐Herrera *et al*. 2019).

The Standardized Precipitation Evapotranspiration Index (SPEI) describes drought conditions based on a balance of precipitation and potential evapotranspiration (Vicente‐Serrano *et al*. 2010). Here, we used long‐term SPEI data provided by the Global Drought Monitor at a spatial resolution of 1° from the grid cell closest to our study site (Beguería *et al*. 2010). The 3‐month aggregated SPEI reaches a minimum value of −1.86 in October 2017 (see Fig. S1), describing a severe drought (McKee *et al*. 1993). Aggregated across 6 months, the SPEI is lower, with a minimum of −2.5 in October 2017. This indicates extreme drought conditions (McKee *et al*. 1993), with 6‐months SPEI being continuously lower than −2 between September and December 2017. Thus, different time scales suggest different drought categories based on McKee *et al*. (1993), and in the following we will use the more conservative terminology, describing the drought as a severe event instead of an extreme drought.

**Fig. 2:**
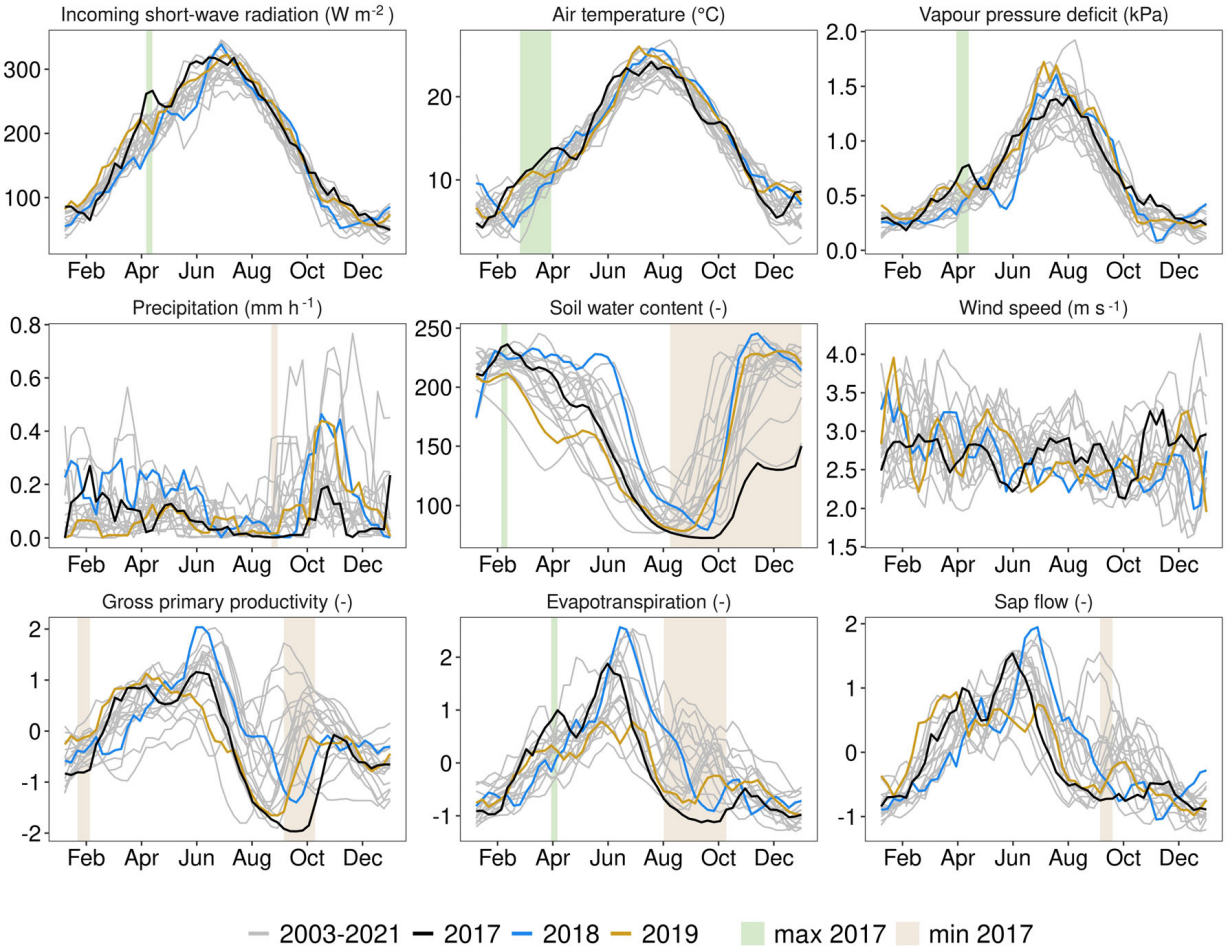
Time evolution of hydro‐meteorological variables and vegetation response. Drought and post‐drought years are shown in coloured lines and all other years in grey lines. Background colours highlight periods when the variable in the drought year has the minimum or maximum value compared to all other years (only if >1 week). For visualization, a 4‐week smoothing is applied in this figure. GPP, ET and sap flow are unitless as a result of data processing that was necessary to obtain a consistent time series across different sensors; the temporal dynamics are consistent across the study period, but absolute values cannot be physically interpreted (see Section 2.3).

In our study site, hydro‐meteorological conditions and ecosystem fluxes showed clear deviations in 2017 compared to other years (Fig. 2). We find the drought to be defined by a severe lack of precipitation, resulting in an annual minimum precipitation for the period 2003 until 2021 (2017: 614 mm, mean 2003–2021: 951 mm) (Fig. S2). The spring of 2017 (February–April) was characterized by warmer temperatures, approximately 2°C above average, and SWC was higher by 5% compared to the average. Between July and September 2017 precipitation reached only 11% of the mean precipitation (2017: 19 mm, mean 2003–2021: 178 mm), which is the lowest precipitation registered between 2003 and 2021. The lack of precipitation resulted in the lowest SWC on our record between August and December 2017. Also, simulated Soil Water Potential and observed pre‐dawn leaf water potential were lowest between September and October (Fig. S3), with minimum pre‐dawn leaf water potential below −5 MPa. A study by Limousin *et al*. (2022) showed that leaf water potential in 2017 at this site exceeded the turgor loss point by 1.4 MPa and was close to a water potential where leaves would lose 50% of hydraulic conductance (P50).

**Fig. 3:**
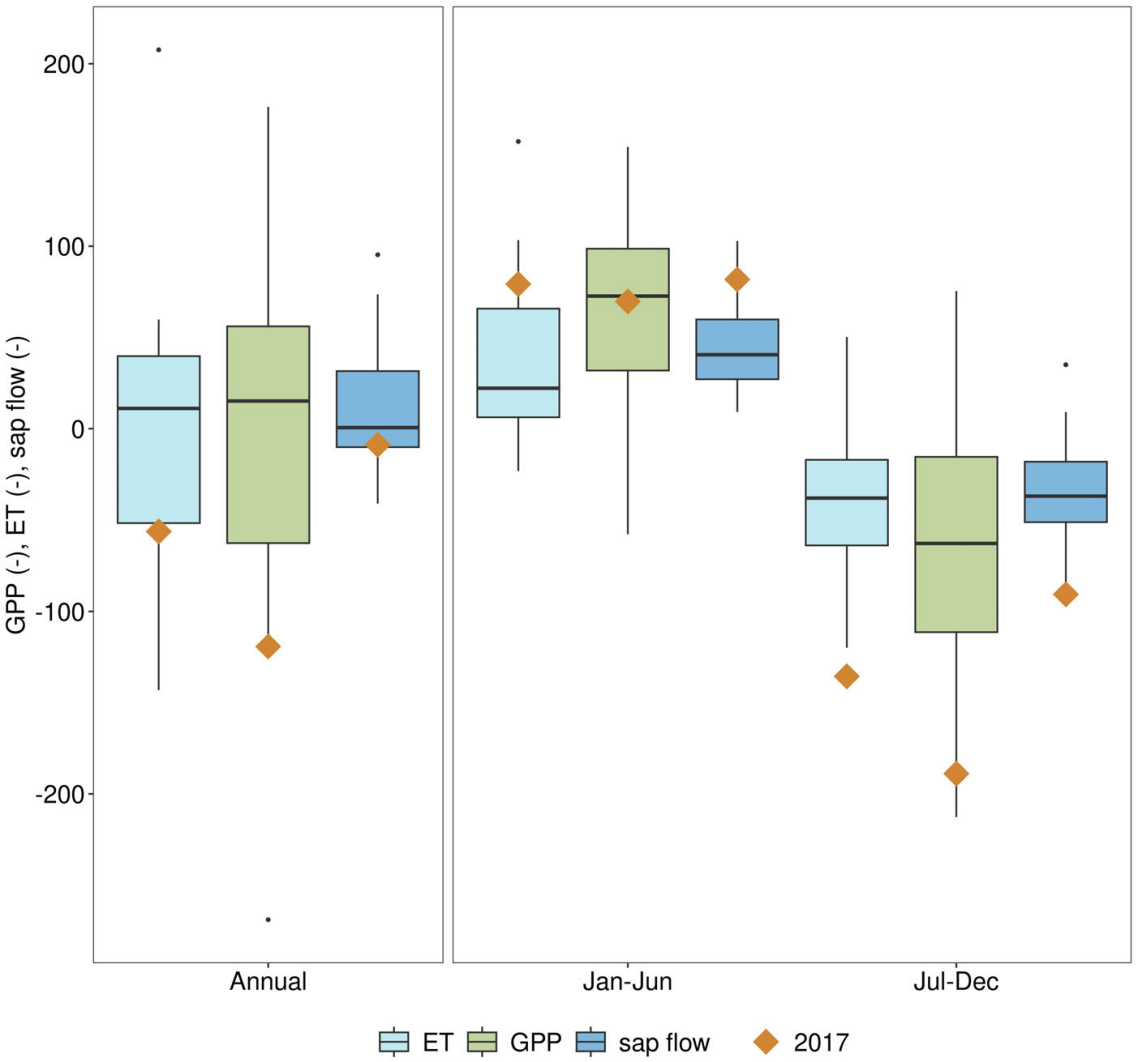
Drought effects on Gross Primary Productivity (GPP), Evapotranspiration (ET) and mean sap flow in 2017. All half‐yearly and yearly values of GPP, ET, and sap flow are included in the boxplots. The highlighted dots represent GPP, ET and sap flow during the drought year 2017.

The severe drought conditions reduced GPP, ET and mean sap flow (Fig. 2). Between August and October 2017 all of those fluxes reached minimum values compared to other years. A shift in GPP, ET and mean sap flow between the first and second half of 2017 was observed (Fig. 3). Due to high TA along with average SWC in the first half of 2017, GPP, ET and sap flow were in the upper 55%, 20% and 15% of all years, respectively. In contrast, ET, sap flow, and GPP strongly reduced during the second half of 2017. The lowest mean ET and sap flow were observed from the studied period of 2003 to 2021, and the second lowest mean GPP. Aggregated across the whole year, GPP, ET and mean sap flow were below average, with GPP showing the strongest response (lowest 10%, 20%, 35% of all years for GPP, ET and sap flow, respectively).

Additionally, visual inspection supports strong drought conditions in Puéchabon in 2017. A study by Ruffault *et al*. (2023) used a plant‐hydraulics model to describe leaf moisture content and found a loss of conductance in the leaves of about 20% in 2017. Simultaneously, canopy moisture content was reduced, and structural damage was observed as leaf desiccation increased (see also their Fig. 5). Enhanced leaf shedding is a sign of severe drought stress in evergreen oaks, such as *Q. ilex* (Barbeta & Peñuelas 2016).

### Post‐drought conditions and ecosystem recovery

The drought in the Mediterranean basin was ended abruptly by a sudden stratospheric warming in early 2018 that caused the largest rainfall anomalies since 1979 in parts of the Iberian Peninsula (Ayarzagüena *et al*. 2018). The year 2018 registered the highest annual precipitation in our study period (1484 mm; Fig. S2). These wet conditions contrasted with the extreme drought conditions in Central Europe in 2018 (Schuldt *et al*. 2020). In Puéchabon, VPD and SW_IN were lower by 18% and 12%, respectively, compared to other years between January and May 2018. Simultaneously, SWC was above average, showing 10% higher values compared to other years (see Fig. 2 also Fig. S4 highlighting extreme values in 2018 and 2019). This resulted in the highest GPP, ET and sap flow in summer 2018 compared to other years in our record.

Thereafter, in 2019 a dry period occurred, with VPD being higher by 12% and SWC by 14% between March and July (Fig. 2). However, dry conditions in summer 2019 were less severe than in summer 2017 in terms of water potentials (Fig. S3). The resulting sap flow was highest compared to all other years between February and March 2019, but maximum values were reached earlier and in summer all fluxes showed a response to dry conditions.

**Fig. 4:**
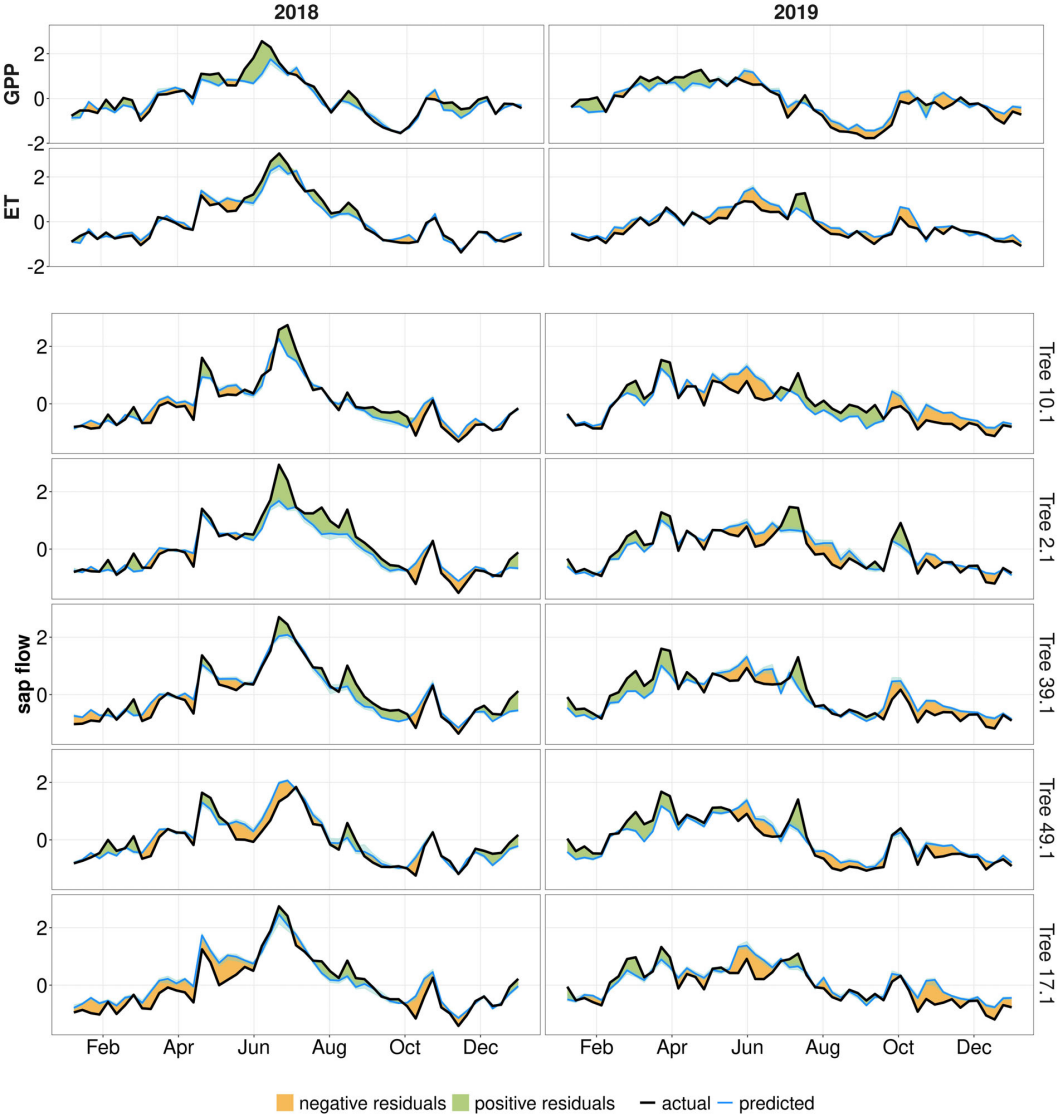
Predicted and observed GPP, ET, and sap flow of individual trees in post‐drought years 2018 and 2019. Random forest prediction shown in blue, actual values in black lines. All time series are processed (see Section 2.3.1) to ensure temporal consistency, while absolute values cannot be physically interpreted. Note that GPP and ET are ecosystem‐scale EC measurements while sap flow is measured at tree‐level.

To assess ecosystem drought recovery, we compare observed fluxes with the predictions of the random forest model based on post‐drought hydro‐meteorological conditions, where deviations can indicate drought legacy effects. Random forest model predictions and observations of GPP, ET and sap flow show overall a high agreement in 2018 and 2019 (Fig. 4). In GPP no consistent deviations were observed within both post‐drought years, and deviations over short time periods were only found in summer 2018 with higher observed than predicted GPP. ET showed varying deviations in 2018. Between April and September 2019, the random forest model overestimated ET for most of the time, except for a short period in July when a peak in ET was observed but not predicted.

In terms of sap flow, we found a strong agreement in the observed and predicted values in most of the trees. Additionally, there was a strong similarity between the trees, which indicates that different trees in the forest experienced similar conditions and responded similarly. Furthermore, sap flow of individual trees showed similar temporal variability as GPP and ET from EC measurement. This indicates that the ecosystem‐scale signals are contributed equally by individual trees.

The peak sap flow in 2018 was underestimated by the random forest model in nearly all trees. Deviations were also found in spring 2018, when sap flow was observed to be higher than expected in some trees. In 2019, model predictions deviated but showed both higher and lower observations than predictions for sap flow.

**Fig. 5:**
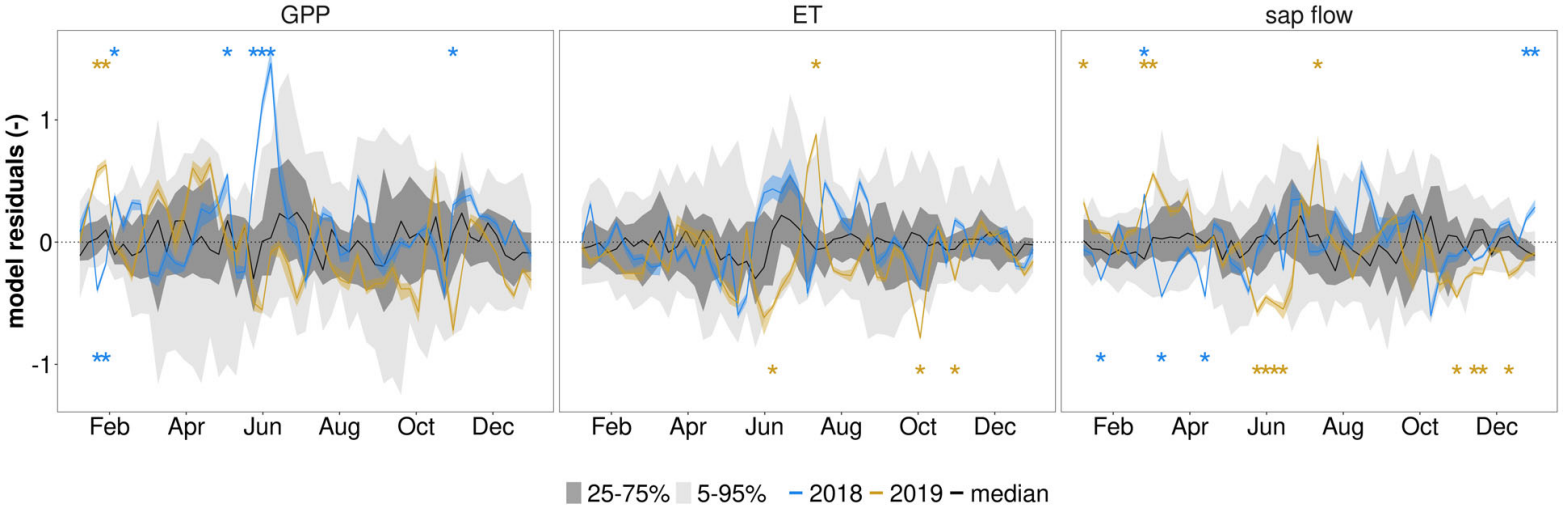
Model residuals across GPP, ET, and sap flow. Residuals of model predictions for post‐drought years (2018–2019) are shown in coloured lines. Black line depicts the median, dark grey area shows the 25th–75th percentile, and light grey area shows the 5th–95th percentile of model uncertainty. Stars indicate weeks when model residuals are outside of the 5th–95th percentile range. Mean OOB scores (‘pseudo R‐squared’) of the underlying random forest models are 0.88, 0.92 and 0.94 for GPP, ET, and sap flow, respectively. Mean root mean squared errors (RMSE) obtained in the leave‐one‐out approach were 0.40, 0.32, and 0.36 for GPP, ET, and sap flow, respectively.

In the next step, we investigated the occurrence of potential legacy effects on GPP, ET, and sap flow. In other words, we analysed if the detected residuals (Fig. 4) were related to legacy effects or to model uncertainty. To this end, we compared the model residuals for the post‐drought years with the model uncertainty (see 2.5) (Fig. 5). Note that in contrast to Fig. 4, the results for sap flow here are based on a random forest model that was trained on all trees jointly to enhance comparability between sap flow and EC based observations. The model residuals are considered significant when they are above or below 95th or 5th percentile of model uncertainty, respectively.

Overall, we found no consistent significant deviation between observations and model predictions, neither in 2018 nor 2019. This was consistent across all studied variables and indicates that the trees fully recovered from the 2017 drought. Additionally, we obtained model residuals of Water Use Efficiency (WUE) calculated by GPP divided by ET from EC measurements (Fig. S5). Concurrently, for WUE we found no systematic legacy effects. Our findings suggest that *Q. ilex* forests can recover fully even from severe droughts such that their physiological functioning is not impaired beyond the duration of the actual water deficit. This might also imply that the severe water deficit in 2017 caused mainly a physiological response, but morphology of *Q. ilex* remained largely intact, apart from increased leaf desiccation.

### Vegetation greenness in drought and post‐drought period

**Fig. 6:**
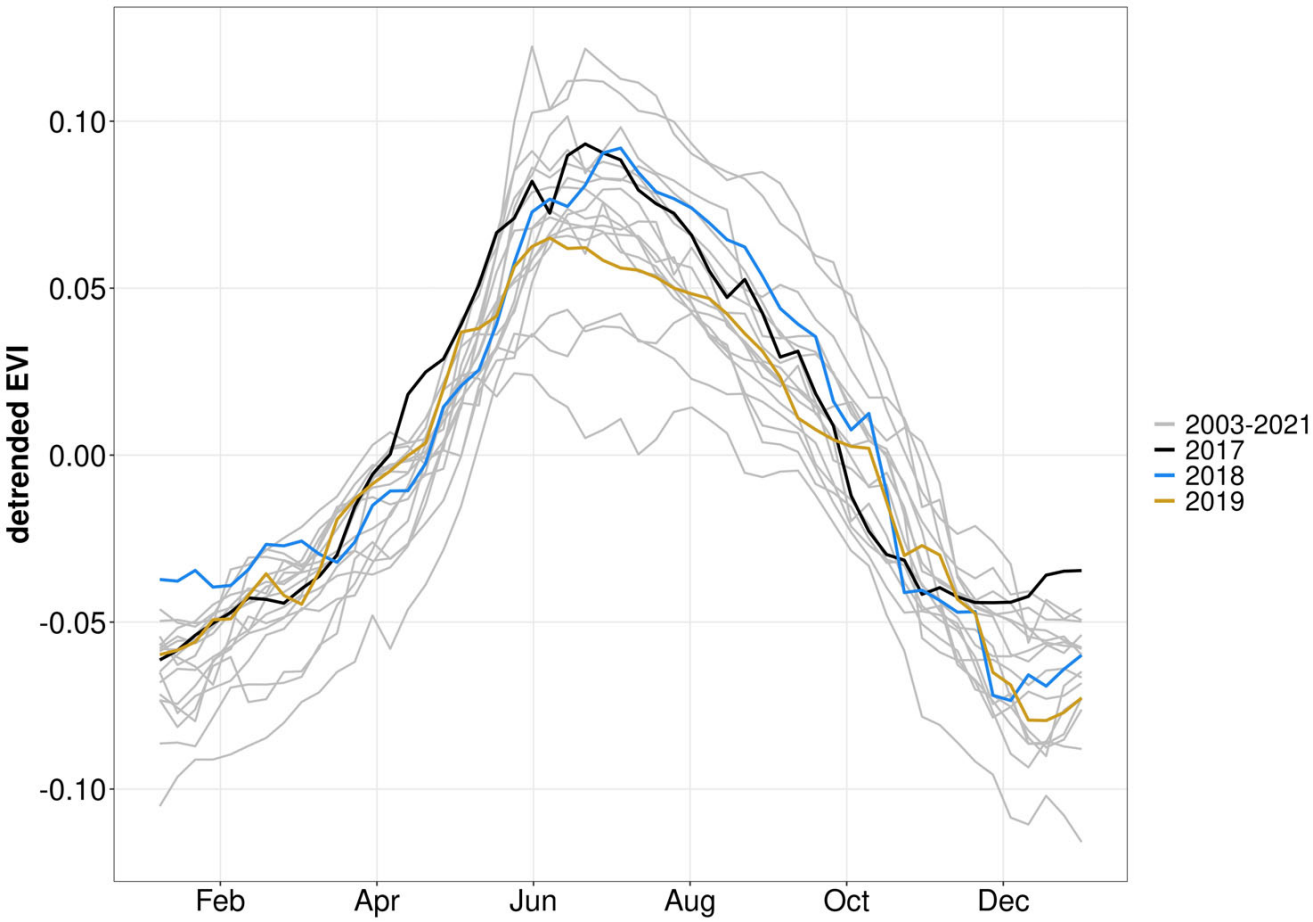
Detrended Satellite‐based Enhanced Vegetation Index (EVI) at Puéchabon between 2003 and 2021. Weekly aggregated data from 2003 to 2021 are shown, with grey colours for 2003–2016 and 2020–2021. 2017–2019 is shown in coloured lines. Note that these data were retrieved from satellite observations such that they represent a larger area than the EC and sap flow measurements. To account for a significant greening trend between 2003 and 2021, a linear trend was subtracted from the original time series.

In addition to ecosystem fluxes, MODIS Enhanced Vegetation Index (EVI, Fig. 6) can reflect vegetation status and potential impacts on aboveground carbon stocks. In the drought year 2017 an earlier increase of vegetation greenness in the first 6 months was observed, which is in line with high GPP supported by warmer‐than‐usual conditions (Fig. 2). In the second half of the year there was a stronger decrease compared to other years, mostly between September and October. This indicates the response of vegetation greenness to the drought. After the drought had ceased, 2018 showed no clear deviations from other years. In the warm and dry year 2019, EVI was relatively low and peaked already in June. In 2019, an earlier decrease was observed, which is comparable to the drought year 2017. This reduced EVI in summer shows a response to the dry period in spring 2019 and corresponds with negative residuals in June 2019 observed in ET, sap flow, and WUE.

We also repeated the random forest analysis for legacy effects for EVI (Fig. S6). Similarly, we find no consistent deviation from predicted values in 2018 and 2019. Thus, the EVI data and analysis confirms the complete recovery of vegetation functioning at this site.

### Importance of pre‐ and post‐drought climate conditions

*Quercus ilex*, as a drought adapted species, was able to fully recover its physiological functioning even after the severe drought of 2017. Negative drought legacy effects might be caused even in highly drought‐adapted species when structural damage occurred (e.g. increased leaf‐shedding as observed in 2017) (Galiano *et al*. 2012; Barbeta & Peñuelas 2016). Thus, we expect that the full recovery observed after the drought in 2017 was supported by beneficial hydro‐meteorological conditions before and after the drought. In early 2017, increased TA and SW_IN along with normal SWC resulted in high GPP. Such pre‐drought conditions can support recovery, when warmer‐than‐usual spring conditions triggered increased productivity which could, in turn, partly compensate for decreased productivity during the drought (Bastos *et al*. 2020; Buermann *et al*. 2018; Wang *et al*. 2020). In Puéchabon, annual stem growth is strongly related to the duration of stem growth in spring and thus higher spring temperatures might allow an increased stem growth (Lempereur *et al*. 2015). However, other studies reported soil moisture deficits in summer to be exacerbated as high temperatures in spring increase evaporation (Lian *et al*. 2020; Li *et al*. 2023). Thus, increased spring temperatures might positively affect stem growth and productivity but, at the same time, increase the water deficit in summer.

After the drought, SWC was quickly refilled in early 2018 by abundant precipitation which prevented persistent soil drought conditions and related stress. Our results are in line with findings at other sites which showed that vegetation recovery was supported by subsequent wet conditions (Jiao *et al*. 2021; Yao *et al*. 2023) or delayed by subsequent dry conditions (Serra‐Maluquer *et al*. 2021; Müller & Bahn 2022; Yao *et al*. 2023).

### Random forest model robustness and application

In this study we characterized drought recovery using a random‐forest approach and detected potential legacy effects based on the comparison of observed and modelled values. Challenges of this approach could arise from both measurement uncertainties and random forest model performances.

Long‐term sap flow measurements as applied here (up to 20 years) were affected by degradation of the measured signal, as tree growth can detach the sensor from the xylem (Moore *et al*. 2010). This reduction in signal strength happened at different sensors with different rates and at different times. So far, there is no established approach to process affected data to derive a consistent long‐term time series without further information on sensor position (see Larsen *et al*. 2020 with an approach including detailed sensor information in data processing). Here, we developed a comprehensive approach and have tested its robustness by manually removing strongly degrading sensors before the processing.

The results of our analysis rely on random forest model predictions of potential sap flow, ET, GPP and EVI whose performance depends on several aspects. For example, to keep the number of predictors low to reduce collinearities in the model, we disregarded effects such as vegetation phenology or preceding meteorological conditions (even though this is indirectly represented through soil moisture data). However, our model uncertainty approach allows for separating significant effects from random variability, thus these shortcomings should not affect our conclusions.

Random forest models have strong ability to account for interacting predictors and produce reliable predictions, but they also have the drawback of being less interpretable compared to other models, such as generalized additive models. However, in this study we use the model mainly to obtain predictions, but do not aim at explaining the drivers of the response. Thus, random forest models were selected for this application.

We evaluated the robustness of our results in different ways. The out‐of‐bag scores (‘pseudo R‐squared’, range 0–1) are 0.88, 0.92 and 0.94 for GPP, ET, and aggregated sap flow, respectively. These high explained fractions of variance indicate the usefulness of the fitted random forest models. The root mean squared error was obtained using the leave‐one‐out approach and resulted in 0.40, 0.32 and 0.36 for GPP, ET, and sap flow, respectively. Also, the random forest models were able to capture the response to the 2017 drought even when 2017 was not included in the training of the model (Fig. S7), indicating that even under drought conditions the model predictions are close to observations. Thus, under moderate to severe water‐limiting conditions in post‐drought years like 2019, the findings should not be strongly affected by a degraded performance of the random forest model. Additional tests were performed to assess the robustness of our results with respect to several aspects: (i) we studied the role of sap flow measured by individual sensors with strong degradation of the signal over time by removing these data from the random forest analysis, (ii) we added additional predictors to the random forest model such as the month‐of‐year to indicate the time of year and thereby vegetation phenology, and (iii) we used only the first half of the training data (2003–2010) to determine the role of the amount of employed training data and of the time distance between training data and predicted period. All tests yielded comparable results to Figs 4 and 5. This supports the robustness of our findings.

## Conclusions

A severe drought in the Mediterranean basin in 2017 caused strong reductions in primary productivity and transpiration in a *Quercus ilex* forest in southern France. Despite these severe direct drought impacts, we found no drought legacy effects, as sap flow, evapotranspiration, and gross primary productivity were not impacted beyond the duration of the drought itself. These results indicate that the physiological functioning of the vegetation recovered fully and quickly after the drought. This was also confirmed by vegetation greenness, as the post‐drought years showed no deviation from other years.

The joint consideration of multiple ecosystem response variables enabled us to comprehensively analyse and diagnose the complete recovery of physiological functioning after drought. In the case of sap flow, we considered individual trees and found that the observed drought response was largely consistent across them. Further, we could validate the robustness of these results with overall matching Eddy covariance measurements. In order to further deepen the understanding of the ecosystem's drought response, future analyses could focus on additional ecophysiological parameters, such as leaf area, non‐structural carbon levels or stem growth (López *et al*. 2009; Kannenberg *et al*. 2019).

The full recovery of *Q. ilex* illustrates high drought resilience (Lloret *et al*. 2011), confirming earlier studies. This is related to favourable spring growing conditions which supported high ecosystem carbon uptake before the drought, whereas abundant precipitation refilled the soil moisture relatively quickly after the drought. While resilience is often studied at annual time scales (Lloret *et al*. 2011), our random forest approach allows us to analyse resilience at shorter time scales and, additionally, can account for the effect of short‐term hydro‐meteorological conditions. This enables the disentangling of legacy effects related to changed plant functioning, which would reduce resilience.

However, even drought‐adapted species might be at risk when the drought intensity and frequency increase further in a changing climate (e.g. Hartmann *et al*. 2022; Limousin *et al*. 2022), as vegetation resilience to droughts is decreased if a second drought occurs before ecosystems can fully recover from the first drought (Müller & Bahn 2022). In Puéchabon, field observations indicate severe impacts after severe droughts in 2022 and 2023. Thus, the full recovery of *Q. ilex* from the 2017 event reported here does not suggest that climate change may not affect these semi‐arid ecosystems (see for example Galiano *et al*. 2012; Piayda *et al*. 2014; Barbeta *et al*. 2015; Caldeira *et al*. 2015; Haberstroh *et al*. 2021). Instead, it highlights that despite strong direct effects of a drought, legacy effects do not necessarily occur, as long as ecosystem recovery is supported by fortunate circumstances, such as favourable pre‐ and post‐drought conditions.

## Author Contributions

AB, RO, CW, SH and XY jointly designed the study. JML provided the site measurements and pre‐processed the data. SH performed the analyses and drafted the article. All authors contributed to the interpretation of the results and discussion of the manuscript.

## Supporting information


**Fig. S1.** Standardized Precipitation Evapotranspiration Index obtained from the Global Drought monitor for 1980–2021. Black areas represent the period covered by Eddy Covariance and sap flow analysis in this study. Blue area highlights the drought year 2017.
**Fig. S2.** Accumulated precipitation (mm) between 2003 and 2021. Colors indicate the total annual precipitation (mm). 2016–2021 highlighted.
**Fig. S3.** Simulated Soil Water Potential (ψ) between 2003 and 2021. Each year from 2003 to 2021 is shown, with grey colors for 2003–2016 and 2020–2021. 2017–2019 in colored lines.
**Fig. S4.** Similar to Fig. 2 but shading indicates extremes observed in 2018 and 2019.
**Fig. S5.** Similar to Fig. 5 but for Water Use Efficiency (WUE). WUE is calculated using Gross Primary Productivity divided by Evapotranspiration.
**Fig. S6.** Similar to Fig. 5 but Enhanced Vegetation Index (EVI).
**Fig. S7.** Similar to Fig. 5 but highlighting model residuals for 2017. For predictions of 2017, the year is excluded from the training.

## Data Availability

The eddy covariance data are fully available from the ICOS Carbon Portal as a FLUXNET Archive (https://data.icos‐cp.eu/portal/). Sap flow data until 2015 are publicly available in the SAPFLUXNET database (https://doi.org/10.5194/essd‐13‐2607‐2021). The Enhanced Vegetation Index is available in the FluxnetEO dataset (https://doi.org/10.5194/bg‐19‐2805‐2022). Any additional data (sap flow data in 2016–2021 and soil moisture and water potential in 2003–2021) are available upon request through the Puéchabon website (https://puechabon.cefe.cnrs.fr/) or the AnaEE ISIA catalogue (https://isia.cnrs.fr/catalog/1/installations/21). The analyses scripts are available online: https://doi.org/10.5281/zenodo.16216807.
