# Supplementary material for: No legacy effects of severe drought on carbon and water fluxes in a Mediterranean oak forest
Source: Plant Biol (Stuttg). 2025 Aug 10;28(3):671–83. doi: 10.1111/plb.70082 (PMC13089598; doi:10.1111/plb.70082)
Supplement: Supplementary file 1 — Fig. S1. Standardized Precipitation Evapotranspiration Index obtained from the Global Drought monitor for 1980–2021. Black areas represent the period covered by Eddy Covariance and sap flow analysis in this study. Blue area highlights the drought year 2017. Fig. S2. Accumulated precipitation (mm) between 2003 and 2021. Colors indicate the total annual precipitation (mm). 2016–2021 highlighted. Fig. S3. Simulated Soil Water Potential (ψ) between 2003 and 2021. Each year from 2003 to 2021 is shown, with grey colors for 2003–2016 and 2020–2021. 2017–2019 in colored lines. Fig. S4. Similar to Fig. 2 but shading indicates extremes observed in 2018 and 2019. Fig. S5. Similar to Fig. 5 but for Water Use Efficiency (WUE). WUE is calculated using Gross Primary Productivity divided by Evapotranspiration. Fig. S6. Similar to Fig. 5 but Enhanced Vegetation Index (EVI). Fig. S7. Similar to Fig. 5 but highlighting model residuals for 2017. For predictions of 2017, the year is excluded from the training. [file PLB-28-671-s001.docx]

## Supplementary


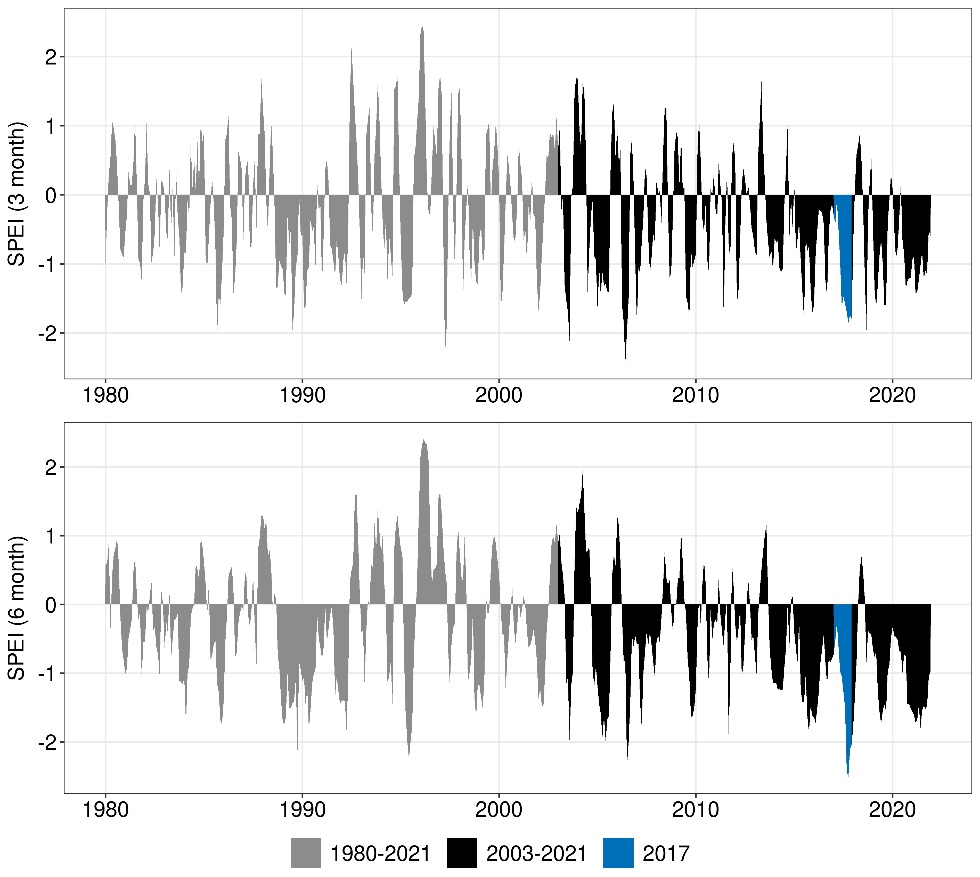


Suppl. Fig. 1: **Standardized Precipitation Evapotranspiration Index obtained from the Global Drought monitor for 1980-2021**. Black areas represent the period covered by Eddy Covariance and sap flow analysis in this study. Blue area highlights the drought year 2017.


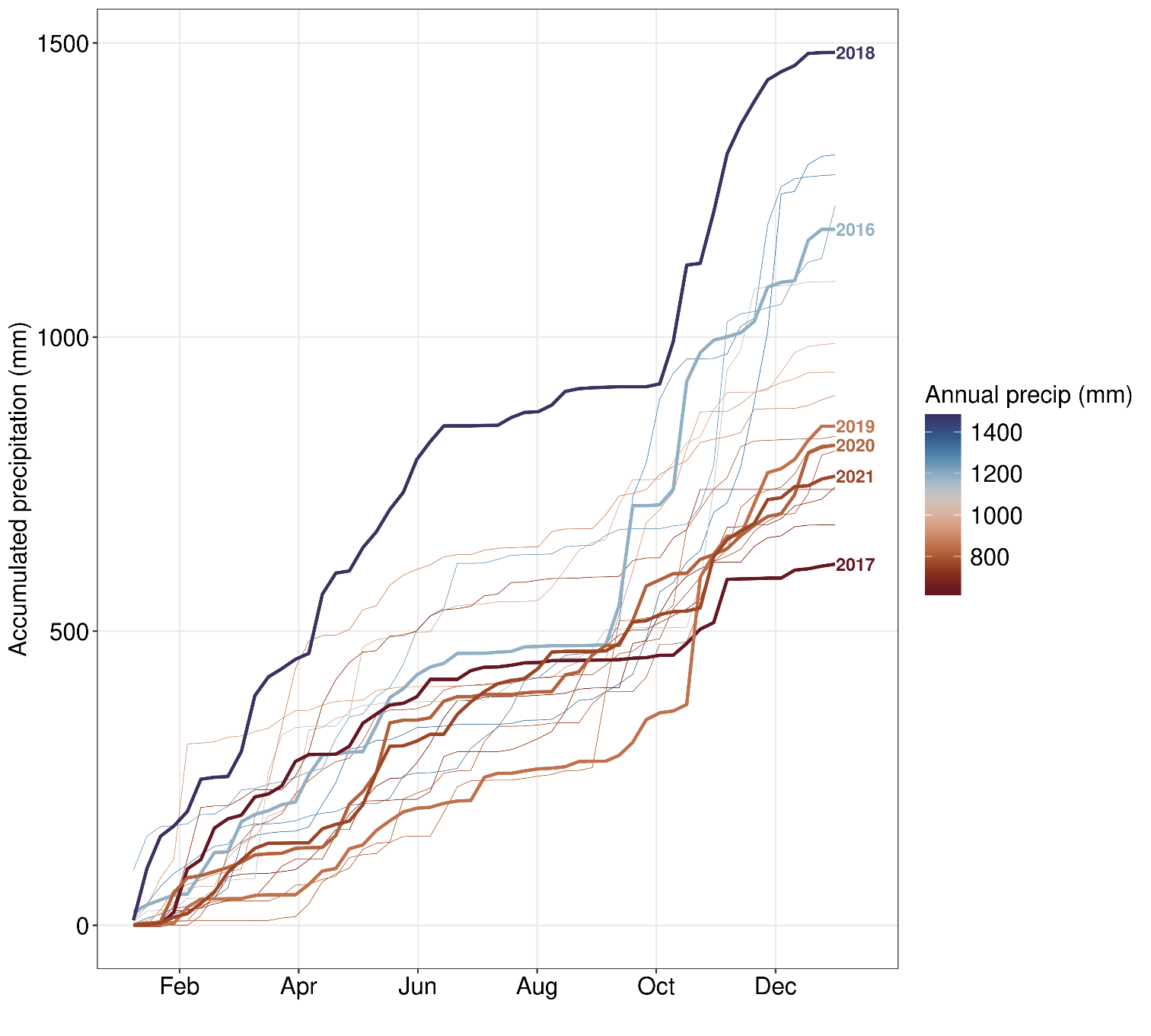


*Suppl. Fig. 2* ***Accumulated precipitation (mm) between 2003 and 2021****. Colors indicate the total annual precipitation (mm). 2016-2021 highlighted.*


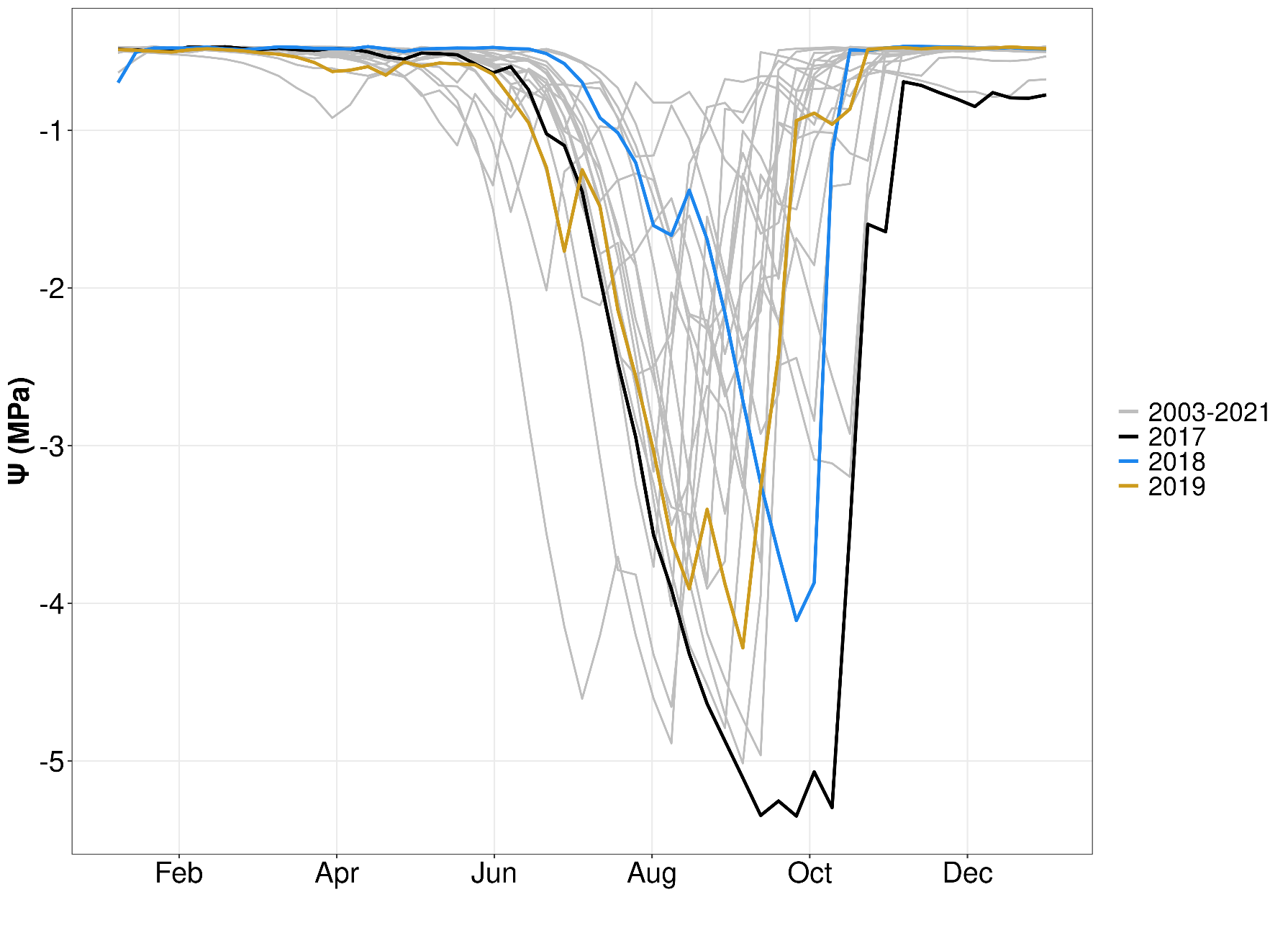


*Suppl. Fig. 3* ***Simulated Soil Water Potential (ψ)*** *between 2003 and 2021. Each year from 2003 to 2021 is shown, with grey colors for 2003-2016 and 2020-2021. 2017-2019 in colored lines.*


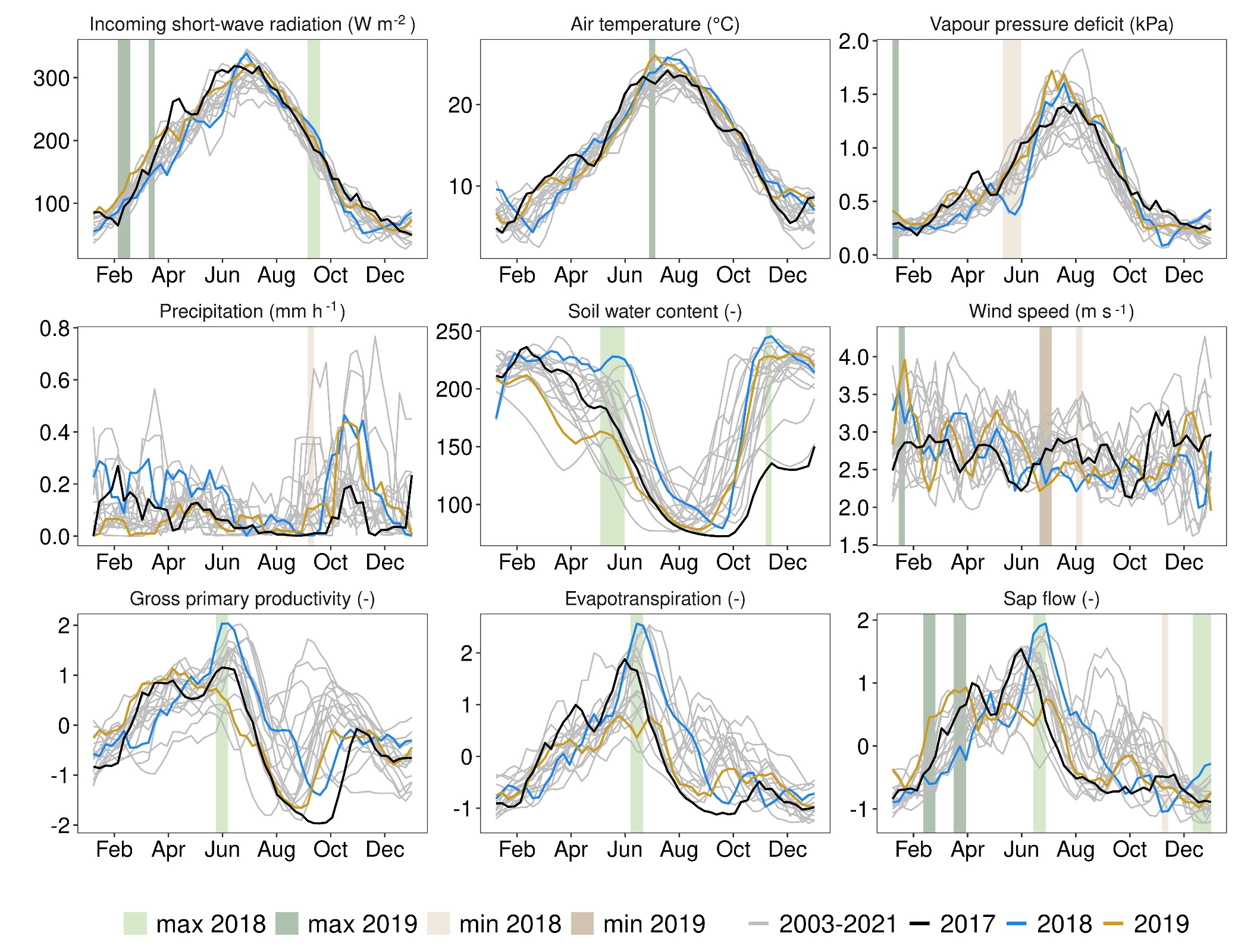


*Suppl. Fig. 4. Similar to Fig. 2 but shading indicates extremes observed in 2018 and 2019*

*
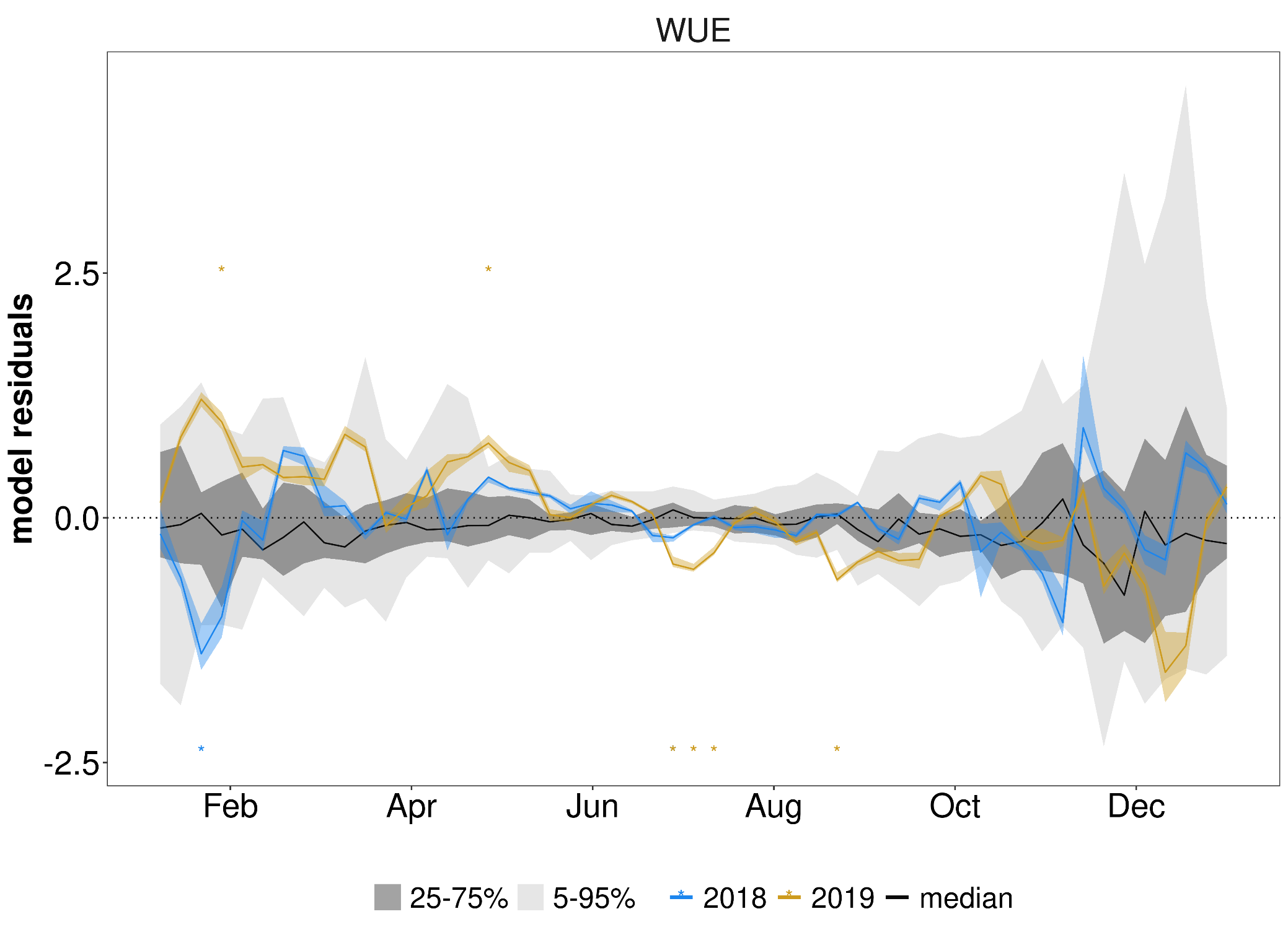
*

*Suppl. Fig. 5. Similar to Fig. 5 but for Water Use Efficiency (WUE). WUE is calculated using Gross Primary Productivity divided by Evapotranspiration.*


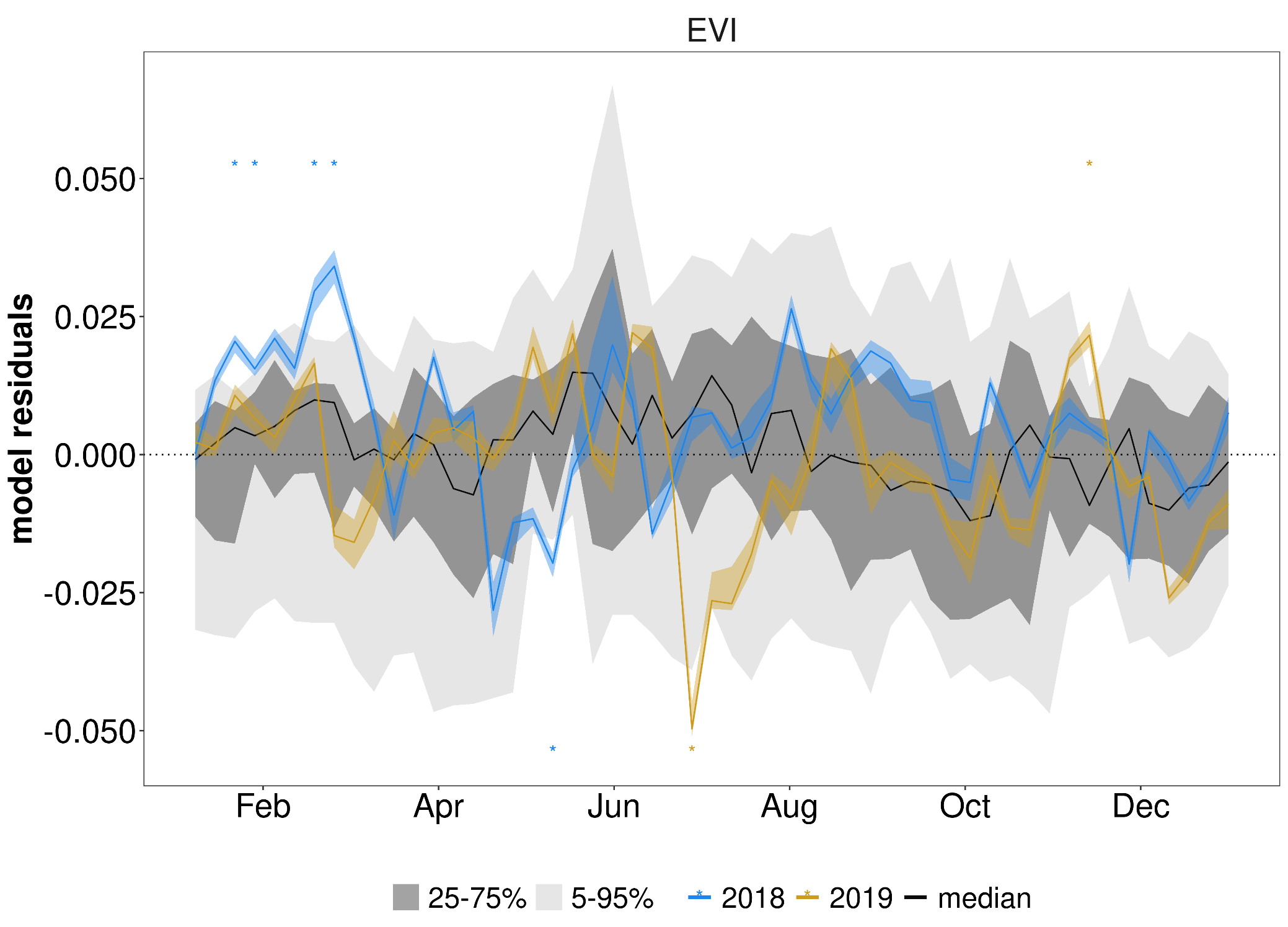


*Suppl. Fig. 6. Similar to Fig. 5 but Enhanced Vegetation Index (EVI).*


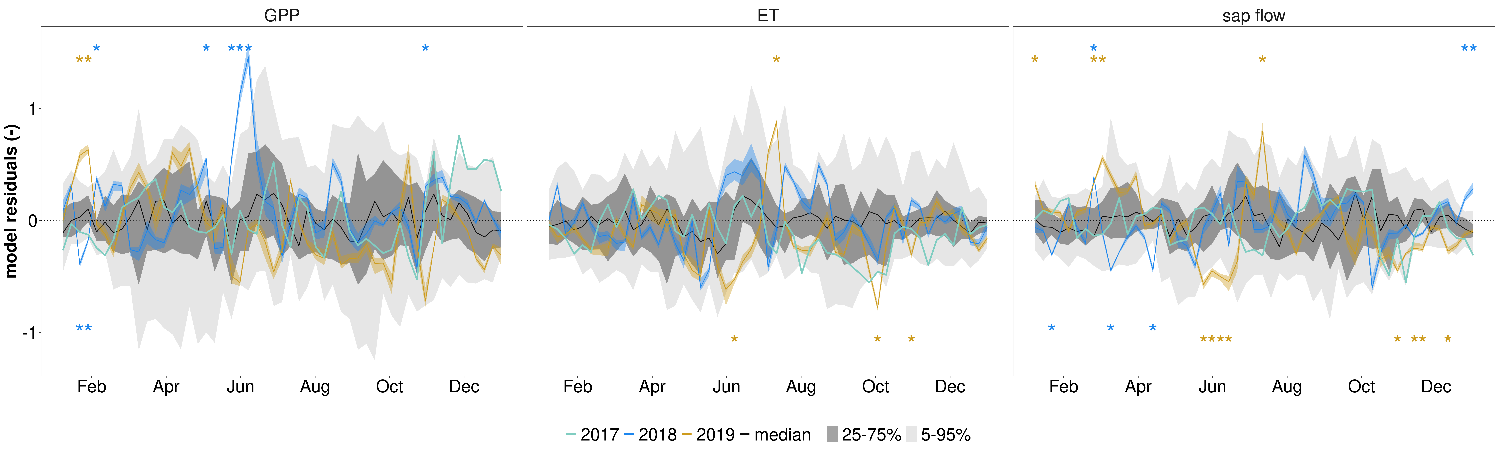


*Suppl. Fig. 7 Similar to Fig. 5 but highlighting model residuals for 2017. For predictions of 2017, the year is excluded from the training.*
